# DPP-4 enzyme deficiency protects kidney from acute ischemia-reperfusion injury: role for remote intermittent bowel ischemia-reperfusion preconditioning

**DOI:** 10.18632/oncotarget.18962

**Published:** 2017-07-04

**Authors:** Yen-Ta Chen, Christopher Glenn Wallace, Chih-Chao Yang, Chih-Hung Chen, Kuan-Hung Chen, Pei-Hsun Sung, Yung-Lung Chen, Han-Tan Chai, Sheng-Ying Chung, Sarah Chua, Fan-Yen Lee, Sheung-Fat Ko, Mel S. Lee, Hon-Kan Yip

**Affiliations:** ^1^ Division of Urology, Department of Surgery, Kaohsiung Chang Gung Memorial Hospital and Chang Gung University College of Medicine, Kaohsiung, Taiwan; ^2^ Department of Plastic Surgery, Royal Devon and Exeter Hospital, Exeter, United Kingdom; ^3^ Division of Nephrology, Department of Internal Medicine, Kaohsiung Chang Gung Memorial Hospital and Chang Gung University College of Medicine, Kaohsiung, Taiwan; ^4^ Division of General Medicine, Department of Internal Medicine, Kaohsiung Chang Gung Memorial Hospital and Chang Gung University College of Medicine, Kaohsiung, Taiwan; ^5^ Department of Anesthesiology, Kaohsiung Chang Gung Memorial Hospital and Chang Gung University College of Medicine, Kaohsiung, Taiwan; ^6^ Division of Cardiology, Department of Internal Medicine, Kaohsiung Chang Gung Memorial Hospital and Chang Gung University College of Medicine, Kaohsiung, Taiwan; ^7^ Division of Thoracic and Cardiovascular Surgery, Department of Surgery, Kaohsiung Chang Gung Memorial Hospital and Chang Gung University College of Medicine, Kaohsiung, Taiwan; ^8^ Department of Radiology, Kaohsiung Chang Gung Memorial Hospital and Chang Gung University College of Medicine, Kaohsiung, Taiwan; ^9^ Department of Orthopedics, Kaohsiung Chang Gung Memorial Hospital and Chang Gung University College of Medicine, Kaohsiung, Taiwan; ^10^ Institute for Translational Research in Biomedicine, Kaohsiung Chang Gung Memorial Hospital and Chang Gung University College of Medicine, Kaohsiung, Taiwan; ^11^ Center for Shockwave Medicine and Tissue Engineering, Kaohsiung Chang Gung Memorial Hospital and Chang Gung University College of Medicine, Kaohsiung, Taiwan; ^12^ Department of Nursing, Asia University, Taichung, Taiwan; ^13^ Department of Medical Research, China Medical University Hospital, China Medical University, Taichung, Taiwan

**Keywords:** acute kidney ischemia-reperfusion injury, preconditioning, inflammation, oxidative stress, dipeptidyl peptidase 4 deficiency

## Abstract

We analyzed the effects of acute ischemia-reperfusion (KIR) injury on the status of kidney function and architecture in dipeptidyl peptidase4-difficient (DPP4^D^) rats and the effect of remote small bowel ischemia-reperfusion (BIR) preconditioning. DPP4-deficient (DPP4^D^) and normal Fischer344 (F344) rats were divided into 6 groups: (1) sham-F344, (2) sham-DPP4^D^, (3) KIR-F344 (4) KIR-DPP4^D^, (5) DPP4^D^-KIR-extendin-9-39 and (6) BIR-KIR-F344. Blood creatinine and urea nitrogen levels and the urinary protein-to-creatinine ratio was higher in KIR-F344 rats than BIR-KIR-F344 or KIR-DPP4^D^ rats 72 h after acute KIR. Conversely, the circulating glucagon-like peptide 1 (GLP-1) levels were higher in BIR-KIR-F344 and KIR-DPP4^D^ than KIR-F344 rats after acute KIR. KIR-F344 rats showed greater inflammation, oxidative stress, apoptosis, DNA damage and kidney injury than other rat groups. Damage to the kidney architecture in KIR-F344 rats was greater than in BIR-KIR-F344 or KIR-DPP4^D^ rats. Expression of antioxidant proteins and GLP-1 receptor was higher in kidneys from KIR-DPP4^D^ and BIR-KIR-F344 than KIR-F344 rats, which suggests better intrinsic responses. We therefore suggest that elevated circulating GLP-1 levels due to DPP4 deficiency and BIR preconditioning protect kidney function and architecture during acute IR injury.

## INTRODUCTION

Despite novel drugs and improvements in critical care including renal replacement therapy, the outcomes for critically ill acute kidney injury (AKI) patients remains poor and its incidence during hospitalization keeps increasing [1–6]. Acute kidney ischemia-reperfusion (KIR) that causes AKI is frequently observed during contrast-media induced nephropathy [5], post-resuscitation shock [7], kidney transplantation [8] and chemical or drug toxicity. These result in acute tubular-epithelial damage [9, 10], loss of tubular microvasculature [11] and inflammation and leukocyte infiltration [8–10, 12].

Acute KIR injury involves oxidative stress and reactive oxygen species (ROS) [13–15], mitochondrial damage [13–15], apoptosis [11, 13–15], and complement cascade and inflammation [11–15]. Therefore, suppression of inflammation and oxidative stress/ROS may be beneficial for AKI patients.

Glucagon-like peptide-1 (GLP-1) based drugs are emerging therapeutics for type 2 diabetes mellitus (DM). GLP-1 reduces inflammation and oxidative stress/ROS [14, 16–18] and the GLP-1 receptor (GLP-1R) is expressed in the brain, kidney, digestive organs and heart and upregulated during IR injury [14, 19–21]. Therefore, increased circulating levels of GLP-1 may protect the kidney from acute IR injury.

GLP-1 is the substrate of dipeptidyl peptidase 4 (DPP4) or CD26, which is a membrane-anchored ecto-protease that cleaves GLP-1. Hence, pharmacological inhibition of DPP4 enhances circulating GLP-1 levels [21–25] in animal models of acute kidney IR injury [14, 21, 23]. However, the direct association between DPP4 and GLP-1 has not been demonstrated, especially in regard to protection from acute IR injury [14, 15, 21, 24, 25].

The role of ischemic preconditioning in protecting organs from ischemic injury has been the focus of many investigations, especially related to the acute coronary syndrome [26–28]. Studies have shown that remote organ ischemia-related preconditioning protects other organs/tissues against IR injury [29–31]. Especially, intestinal ischemic preconditioning suppresses oxidative stress and protects remote organs from ischemic reperfusion damage [32, 33]. These findings suggest that intermittent small bowel IR (BIR) may protect kidneys from acute IR injury since the gastro-intestinal tract secretes GLP-1 [26–33]. Therefore, in this study, we tested if DPP4 deficiency and intermittent BIR preconditioning protected kidneys against acute IR injury.

## RESULTS

### Circulating creatinine and blood urea nitrogen (BUN) levels and urine protein to creatinine ratio at baseline and day 3 after acute KIR

**Figure 1:**
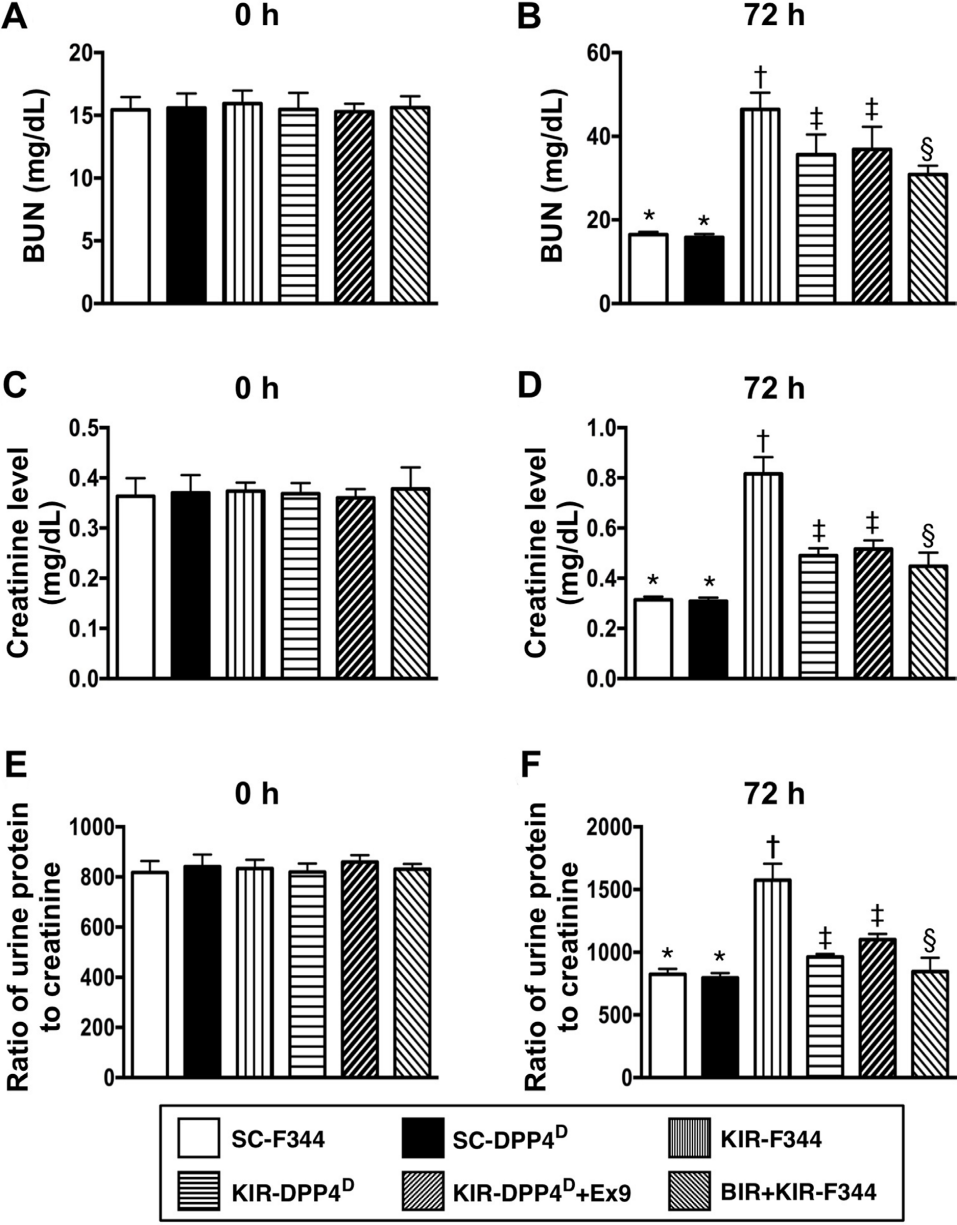
Blood urea nitrogen (BUN) and creatinine levels and urine protein to creatinine ratios at days 0 and 3 after acute KIR (**A**, **B**) Circulating levels of blood urine nitrogen (BUN) at day 0 (*p* > 0.5) and day 3 after acute KIR in the 6 groups of rats. (**C**, **D**) Circulating level of creatinine at day 0 (*p* > 0.5) and day 3 after acute KIR in the 6 groups of rats. (**E**, **F**) Urine protein to creatinine ratio on day 0 (*p* > 0.5) and day 3 after acute KIR in the 6 groups of rats. Note: *denotes statistical significance vs. other groups with different symbols (†, ‡, §); *p* < 0.0001. All statistical analyses were performed by one-way ANOVA and Bonferroni multiple comparison post-hoc test (*n* = 8 for each group). Symbols (*, †, ‡, §) indicate statistical significance. SC = sham control; DPP4^D^ = dipeptidyl peptidase 4 deficient; BIR = small bowel ischemia-reperfusion; KIR = kidney ischemia-reperfusion.

Before the IR procedure, blood urea nitrogen (BUN) and blood creatinine were similar in all 6 groups of rats. Similarly, the protein to creatinine ratios in the urine was similar in the 6 groups of rats (Figure 1). However, 72 h after acute KIR, blood creatinine and BUN levels that indicate renal dysfunction, and the urine protein to creatinine ratio that indicate glomerular damage were lowest in the SC-F344 and SC-DPP4^D^ rats and gradually increased in BIR-KIR-F344, KIR-DPP4^D^, DPP4^D^-KIR- exendin-9-39 rats and were highest in KIR-F344 rats (Figure 1).

### Kidney injury score and circulating GLP-1 levels on day 3 after acute KIR

**Figure 2:**
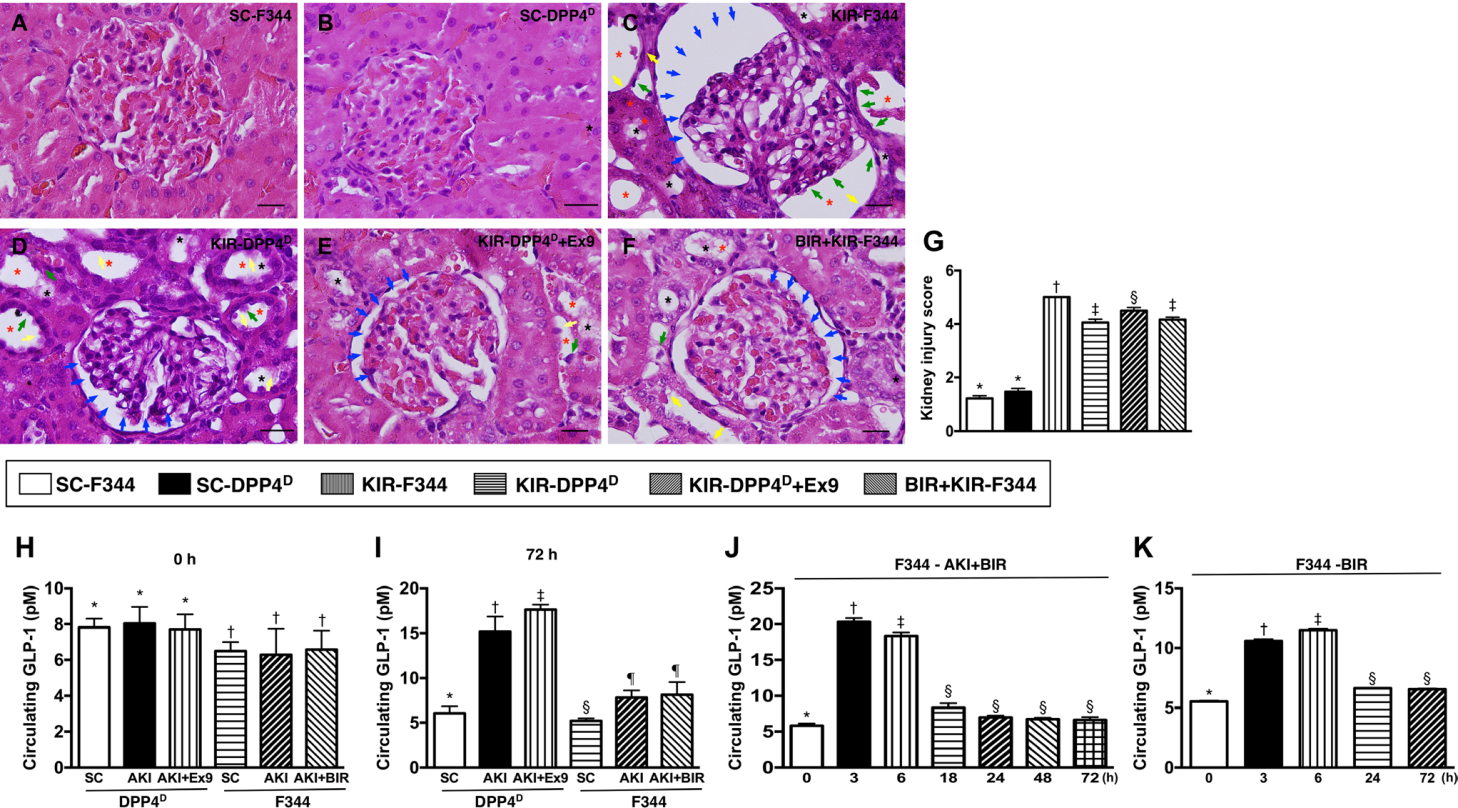
Kidney injury scores and circulating levels of GLP-1 at different time points after acute KIR (**A**–**F**) Representative H&E stained kidney sections (200×) of the 6 groups of experimental rats demonstrating higher loss of brush border in renal tubules (yellow arrows), tubular necrosis (green arrows), tubular dilation (red asterisk), protein cast formation (black asterisk), and dilation of Bowman's capsule (blue arrows). (**G**) Kidney injury scores based on pathological analysis based on H&E sections of the 6 groups of experimental rats is shown. Note: * denotes statistical significance vs. other groups with different symbols (†, ‡, §), *p* < 0.0001. Scale bars in right lower corner represent 20 μm. (**H**) Baseline (day 0) circulating levels of GLP-1 in the 6 rat groups; * denotes statistical significance vs. ^†^*p* < 0.05. (**I**) Circulating levels of GLP-1 in KIR-DPP4^D^ and KIR-F344 group of rats on day 3 after KIR. * denotes statistical significance vs. other groups with different symbols (†, ‡), *p* < 0.0001; § denotes statistical significance vs. ^¶^*p* < 0.01. (**J**) Circulating levels of GLP-1 at different time points in BIR-KIR-F344 animals. * denotes statistical significance vs. other groups with different symbols (†, ‡, §); *p* < 0.0001. (**K**) Circulating levels of GLP-1 at different time points in BIR-F344 animals. *denotes statistical significance vs. other groups with different symbols (†, ‡, §); *p* < 0.0001.

We scored the H&E stained sections for kidney injury and found that SC-F344 and SC-DPP4^D^ rats had lowest scores. The scores were gradually higher for BIR-KIR-F344, KIR-DPP4^D^, DPP4^D^-KIR-exendin-(9-39) rats and were highest in KIR-F344 rats (Figure 2).

The baseline circulating levels of GLP-1 were higher in the SC-DPP4^D^ rats than SC-F344 rats. Further, the circulating levels of GLP-1 on day 3 after acute KIR procedure were significantly lower in the SC-F344 than KIR-F344 and KIR-BIR-F344 group rats, which had similar GLP-1 levels. Circulating GLP-1 levels were higher in the KIR-DPP4^D^ group and highest in KIR-DPP4^D^ + Ex4-9-39 group compared to the SC-DPP4^D^ group. The higher circulating GLP-1 levels on day 3 after acute KIR in the KIR-DPP4^D^ + Ex4-9-39 rats compared to KIR-BIR-F344 rats suggested enhanced intrinsic response to the occupied GLP-1R in kidney parenchyma elicited by Ex4-9-39 (Figure 2).

Time course analysis of circulating GLP-1 levels in BIR-KIR-344 and BIR-344 animals showed that they were markedly upregulated only at 3–6 h after BIR-KIR procedure suggesting that GLP-1 elevation was in response to IR (Figure 2).

### Status of inflammation, apoptosis and oxidative-stress on day 3 after acute KIR

**Figure 3:**
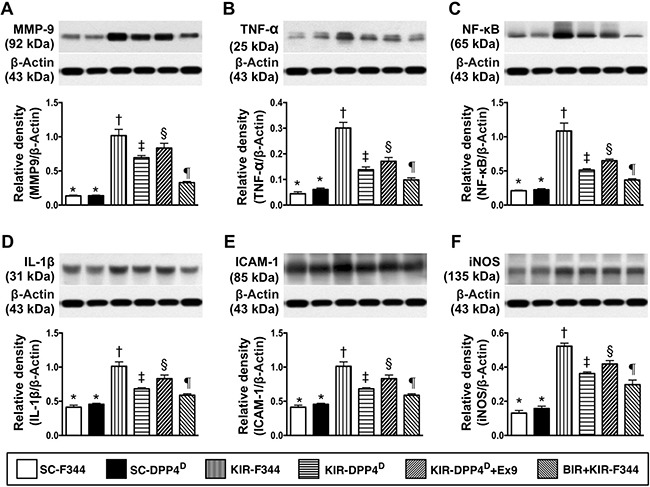
Analysis of kidney inflammation on day 3 after acute KIR Western blotting analysis of (**A**) matrix metalloproteinase-9 (MMP-9), (**B**) tumor necrosis factor-α (TNF- α), (**C**) Nuclear factor-κB (NFκB), (**D**) Interleukin-1β (IL-1β), (**E**) Intercellular adhesion molecule-1 (ICAM-1), and (**F**) inducible nitric oxide synthase (iNOS) in the 6 groups of rats 72 h after acute KIR. Note: *denotes statistical significance vs. other groups represented with different symbols (†, ‡, §, ¶), *p* < 0.0001.

Further, we analyzed the status of inflammation in the 6 groups of mice by analyzing the expression of matrix metalloproteinase (MMP)-9, tumor necrosis factor (TNF)-α, nuclear factor (NF)-κB, interleukin (IL)-1β, intercellular adhesion molecule (ICAM)-1, and inducible nitric oxide synthase (iNOS). We observed that these six indicators of inflammation were lowest in SC-F344 and SC-DPP4^D^ rats and gradually increased in BIR-KIR-F344, KIR-DPP4^D^, DPP4^D^-KIR- exendin-9-39 rats and were highest in KIR-F344 rats (Figure 3).

**Figure 4:**
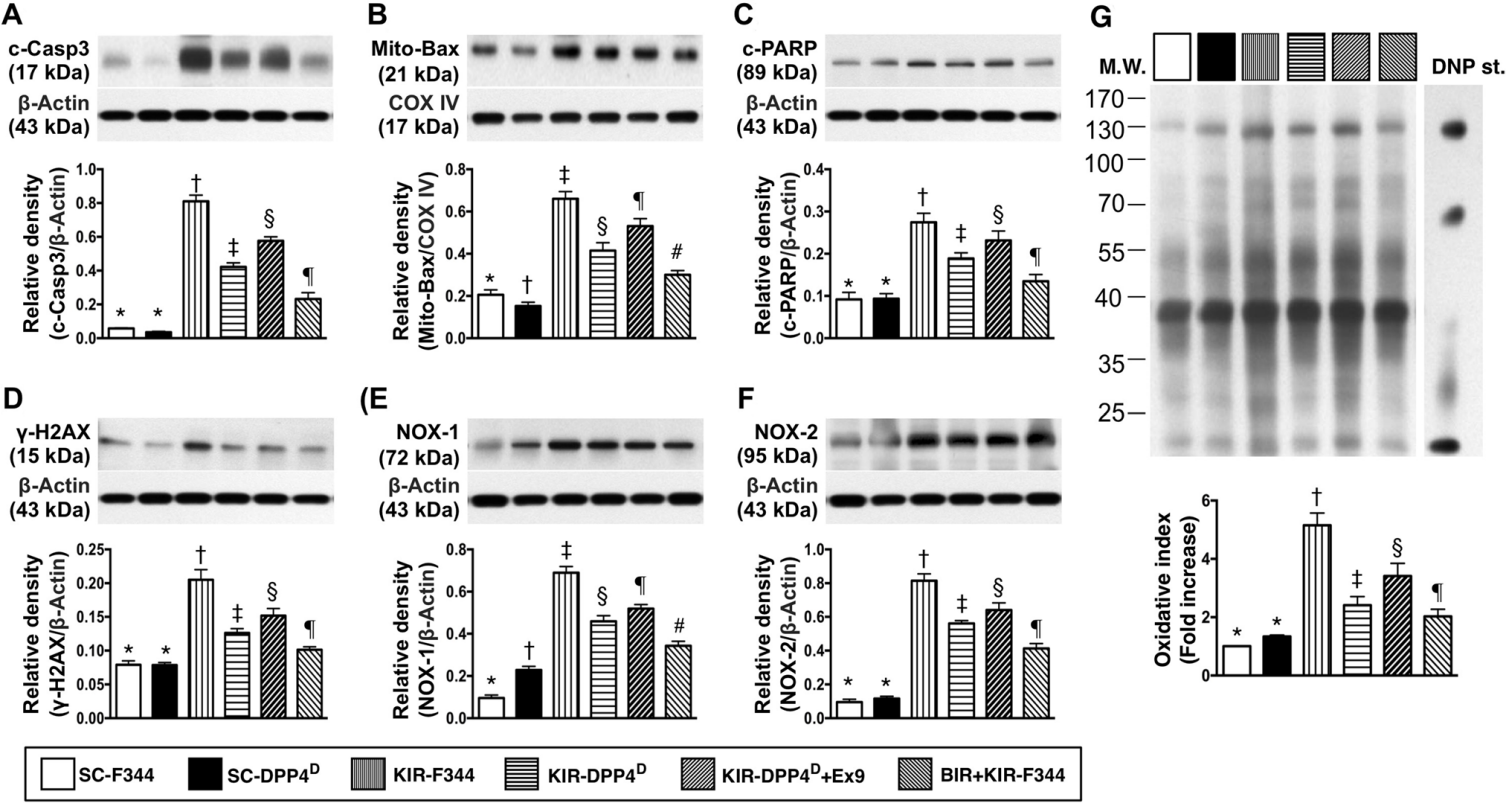
Analysis of apoptotic, DNA damage and oxidative stress in the kidney on day 3 after acute KIR Western blot analysis of (**A**) cleaved caspase 3 (c-Casp3), (**B**) cleaved caspase 3 (c-Casp3), (**C**) cleaved Poly (ADP-ribose) polymerase (c-PARP), (**D**) γ-H2AX, (**E**) NOX-1 and (**F**) total oxidized protein by oxyblot (left most lanes in the upper panel represent protein molecular weight marker and control oxidized molecular protein standard, respectively; DNP is 1-3 dinitrophenylhydrazone) in the 6 groups of rats 72 h after acute KIR. Note *denotes statistical significance vs. other groups represented with different symbols (†, ‡, §, ¶), *p* < 0.0001.

A similar pattern was observed for the 3 apoptotic indicators, namely cleaved caspase 3, cleaved poly (ADP-ribose) polymerase (c-PARP) and mitochondrial-Bax, and γ-H2AX that is a marker for DNA damage and NOX1, NOX2, and total oxidized protein that indicate oxidative stress (Figure 4).

### Status of podocyte components, antioxidants and GLP-R on day 3 after acute KIR

**Figure 5:**
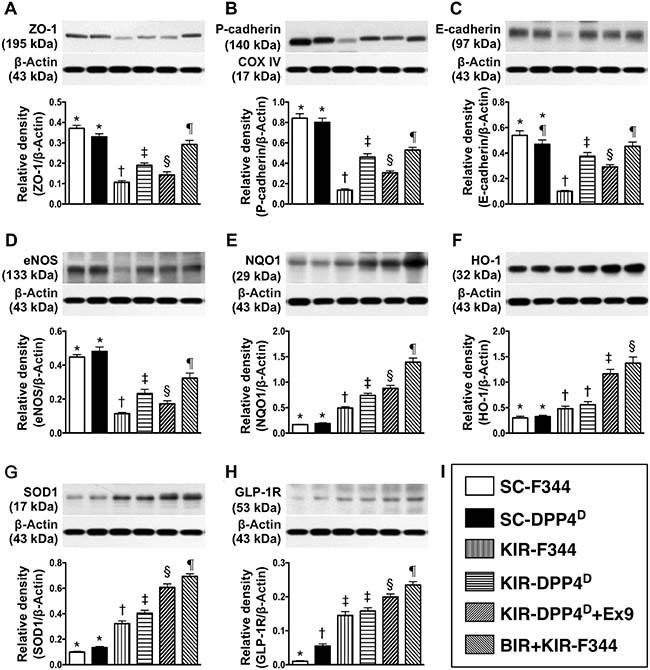
Analysis of podocyte components, antioxidants and GLP-R on day 3 after acute KIR Western blot analysis of (**A**) ZO-1, (**B**) P-cadherin (**C**) E-cadherin (**D**) eNOS, (**F**) NQO1, (**G**) heme oxygenase 1 (HO-1), (**H**) SOD-1 and (**I**) glucagon like peptide receptor (GLP-R) in the 6 groups of rats 72 h after acute KIR. *denotes statistical significance vs. other groups represented with different symbols (†, ‡, §, ¶), *p* < 0.0001.

We observed that zonula occludens 1 (ZO-1), a tight junction-associated protein in podocytes, and P-cadherin and E-cadherin, which are predominantly in renal glomerulus and endothelial nitric oxide synthase (eNOS), an indicator of endothelial function, vasorelaxation integrity and angiogenesis were high in SC-F344 and SC-DPP4^D^ rats and progressively lower in BIR-KIR-F344, KIR-DPP4^D^, DPP4^D^-KIR- exendin-9-39 rats and were lowest in KIR-F344 rats (Figure 5). The levels of GLP-1R and 3 antioxidant proteins, NQO 1, HO-1 and SOD-1 progressively increased from groups 1–6 suggesting an intrinsic response to ischemic stimulation and further upregulation due to DPP4 deficiency and BIR. GLP-1R levels were higher in DPP4^D^-KIR- exendin-9-39 rats compared to KIR-DPP4^D^ suggesting that exendin-9-39 treatment resulted in a competition with GLP-1, thereby enhancing the expression of GLP-1R in kidney parenchyma.

### Status of infiltrating inflammatory cells in kidney parenchyma on day 3 after acute KIR

**Figure 6:**
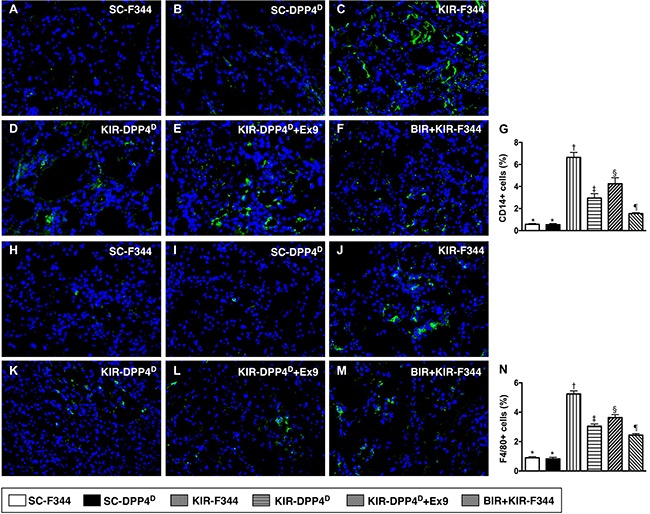
Analysis of infiltration of inflammatory cells in kidney parenchyma on day 3 after acute KIR (**A**–**F**) Representative immunofluorescence (IF) images (400x) showing CD14^+^ cells (green color) in the kidney parenchyma in the 6 groups of rats 72 h after acute KIR. (**G**) Quantification of mean total number of CD14^+^ cells in the kidney sections from 6 groups of rats, 72 h after acute KIR. (**H**–**M**) Representative IF images (400×) showing F4/80+ cells (green color) in kidney parenchyma in the 6 groups of rats 72 h after acute KIR. (**N**) Quantification of mean total number of F4/80+ cells in the kidney sections from 6 groups of rats, 72 h after acute KIR. Note: *denotes statistical significance vs. other groups represented with different symbols (†, ‡, §, ¶), *p* < 0.0001. Scale bars in the right lower corner represent 20 μm. Blue color indicates nuclei were stained by DAPI.

Immunofluorescence (IF) of kidney sections showed low numbers of CD14^+^ and F4/80^+^ cells, two indicators of inflammation in SC-F344 and SC-DPP4^D^ rats and gradually higher in BIR-KIR-F344, KIR-DPP4^D^, DPP4^D^-KIR- exendin-9-39 rats and were highest in KIR-F344 rats (Figure 6).

### Status of cellular oxidative stress and kidney injury on day 3 after acute KIR

**Figure 7:**
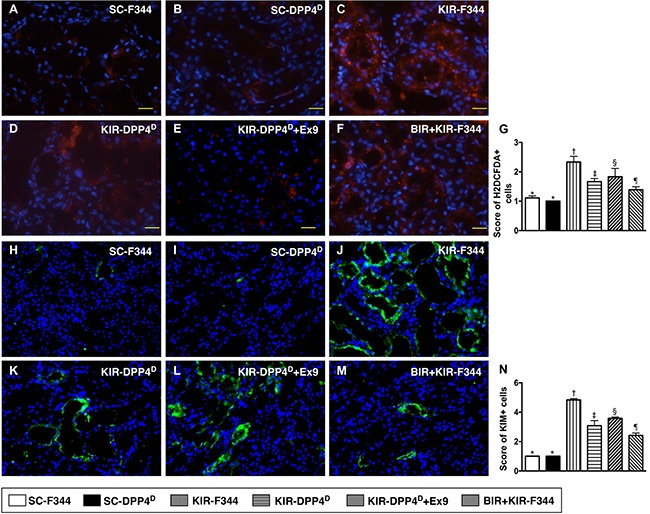
Analysis of cellular oxidative stress and kidney injury markers on day 3 after acute KIR (**A**–**F**) Representative IF images (200×) showing H2DCFDA^+^ cells (red color) in the kidney parenchyma of the 6 groups of rats, 72 h after acute KIR. (**G**) Quantification of total number of H2DCFDA^+^ cells in the kidney sections from 6 groups of rats, 72 h after acute KIR. *denotes statistical significance vs. other groups represented with different symbols (†, ‡, §, ¶), *p* < 0.0001. Scale bars in right lower corner represent 50 μm. (**H**–**M**) Representative IF images (400×) showing KIM^+^- cells (green color). (**N**) Quantification of mean total number of KIM+ cells in the kidney sections from 6 groups of rats, 72 h after acute KIR. *denotes statistical significance vs. other groups represented with different symbols (†, ‡, §, ¶), *p* < 0.0001. Scale bars in right lower corner represent 20 μm. Blue color indicates DAPI stained nuclei.

The SC-F344 and SC-DPP4^D^ rats showed lower numbers of H2DCFDA^+^ cells in the kidney parenchyma, an indicator of ROS, whereas they gradually increased in BIR-KIR-F344, KIR-DPP4^D^, DPP4^D^-KIR- exendin-9-39 rats and were highest in KIR-F344 rats (Figure 7). The expression pattern of KIM-1, a kidney injury biomarker that is predominantly expressed in renal tubules was similar to H2DCFDA in the six rat groups.

### Status of podocyte, extracellular-matrix and renal tubule components on day 3 after acute KIR

**Figure 8:**
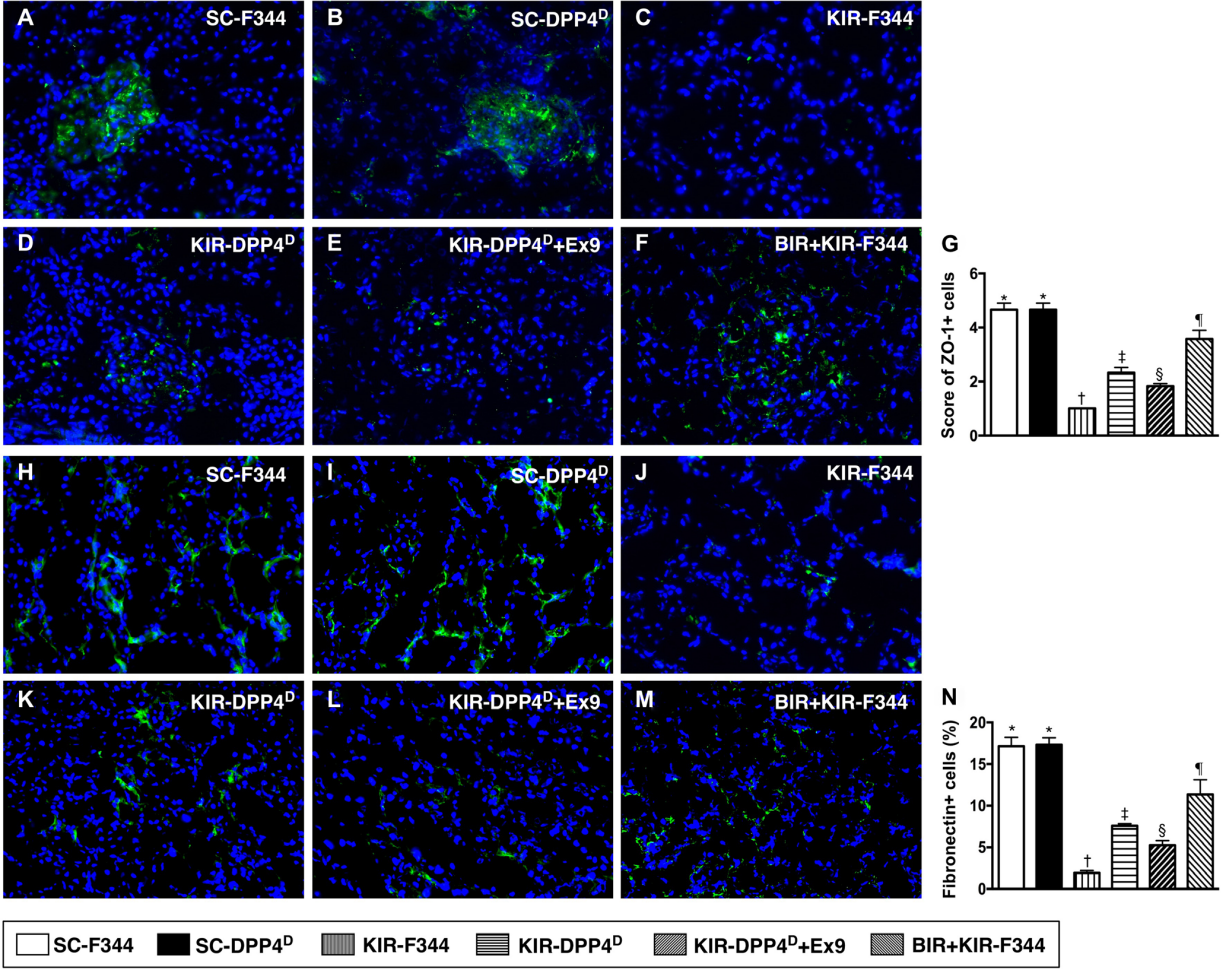
Immunofluorescence (IF) analysis of ZO-1 and fibronectin expression on day 3 after acute KIR (**A**–**F**) Representative IF images (400×) showing positively stained ZO-1 (green color) distribution in glomeruli of the 6 groups of rats, 72 h after acute KIR. (**G**) Quantification of total number of ZO-1 positive cells in the kidneys of 6 groups of rats, 72 h after acute KIR. (**H**–**M**) Representative IF images (400×) showing positively stained fibronectin (green color) distribution in extracellular matrix of 6 groups of rats, 72 h after acute KIR. (**N**) Quantification of total number of fibronectin positive cells in kidneys of the 6 groups of rats, 72 h after acute KIR. *denotes statistical significance vs. other groups represented with different symbols (†, ‡, §, ¶), *p* < 0.0001. Scale bars in right lower corner represent 20 μm. Blue color indicates DAPI stained nuclei.

Both ZO-1, a tight junction-associated protein in podocytes and Fibronectin, which is involved in cell-extracellular matrix interactions were highly expressed in SC-F344 and SC-DPP4^D^ rats and progressively lower in BIR-KIR-F344, KIR-DPP4^D^, DPP4^D^-KIR- exendin-9-39 rats and were lowest in KIR-F344 rats (Figure 8). Fibronectin is situated in the mesangial and interstitial extracellular matrix and defective cell-extracellular matrix interactions contribute to ischemic renal failure [34].

**Figure 9:**
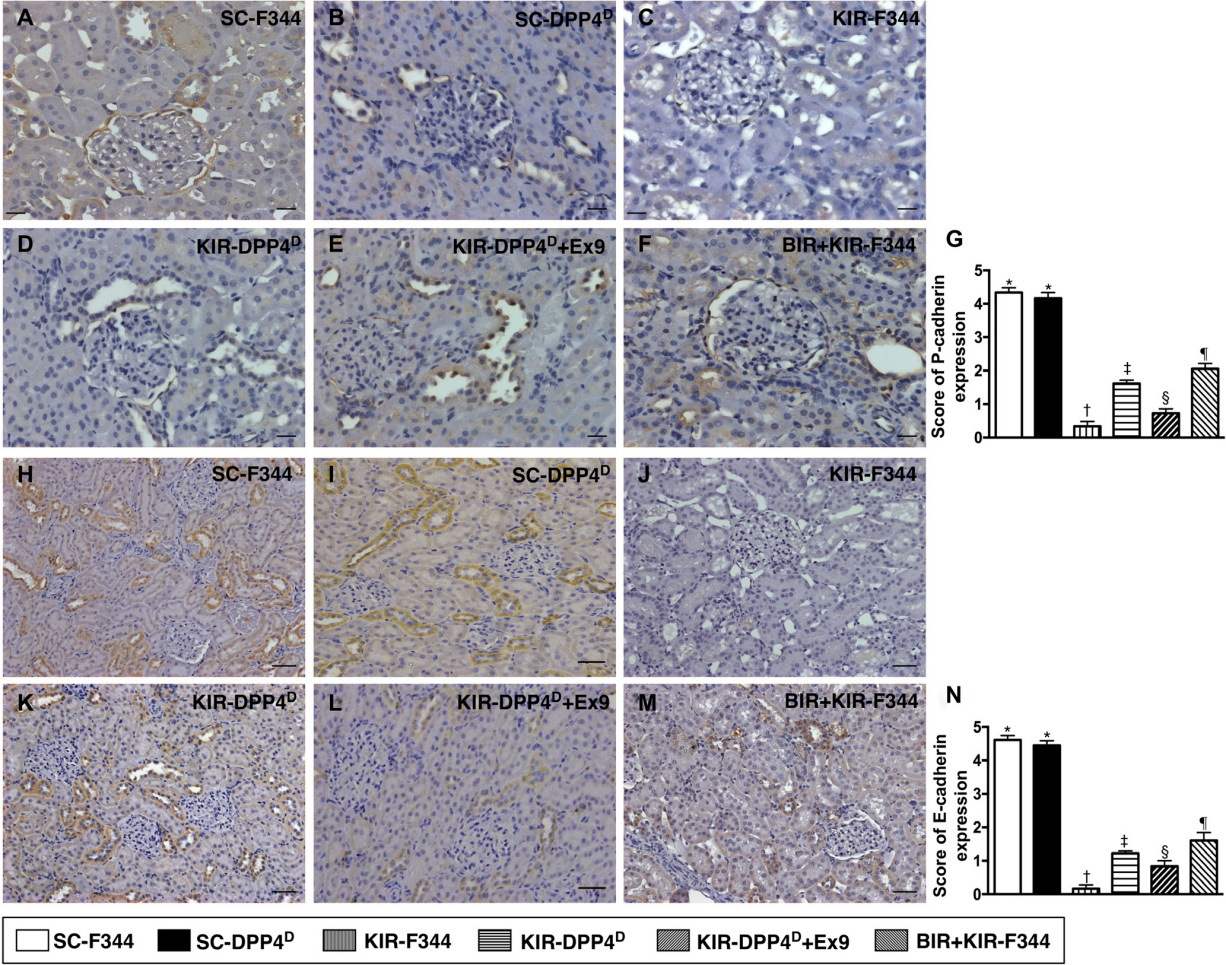
Immunohistochemical (IHC) analysis of P-cadherin and E-cadherin expression on day 3 after acute KIR (**A**–**F**) Representative IHC images (200×) showing positively stained P-cadherin (gray color) distribution in renal tubules of the 6 groups of rats, 72 h after acute KIR. (**G**) Quantification of total number of P-cadherin positive cells in the kidney sections from 6 groups of rats, 72 h after acute KIR injury. (**H**–**M**) Representative IHC images (200×) showing positively stained E-cadherin (gray color) distribution in extracellular matrix of 6 groups of rats, 72 h after acute KIR. (**N**) Quantification of total number of E-cadherin positive stained cells in the kidney sections from 6 groups of rats, 72 h after acute KIR. *denotes statistical significance vs. other groups represented with different symbols (†, ‡, §, ¶), *p* < 0.0001. Scale bars in right lower corner represent 50 μm in the kidney sections from 6 groups of rats, 72 h after acute KIR injury.

We then analyzed the expression of P-cadherin and E-cadherin by IHC, which are Ca^2+^-dependent cell adhesion molecules involved in the development and maintenance of renal epithelial polarity. They showed an identical expression pattern to ZO-1 among the six groups (Figure 9).

**Figure 10:**
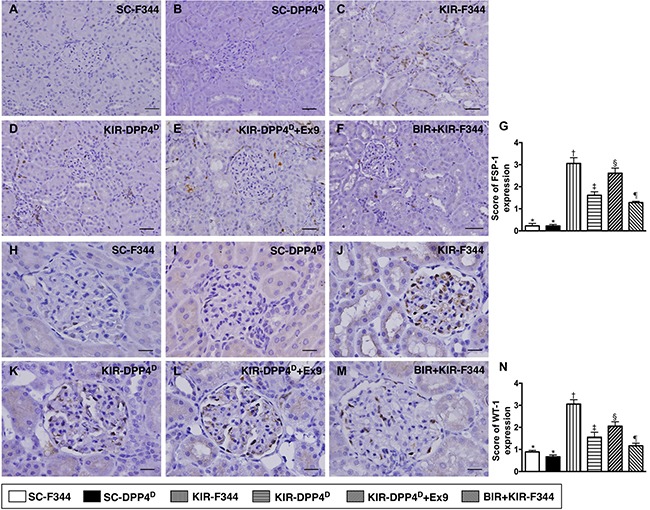
Immunohistochemical (IHC) analysis of FSP-1 and WT-1 expression on day 3 after acute KIR (**A**–**F**) Representative IHC images (200×) showing positively stained fibroblast specific protein-1 (FSP-1) (gray color) distribution in kidney interstitials from 6 groups of rats, 72 h after acute KIR injury. (**G**) Quantification of total number of FSP-1 positive cells in the kidney sections from 6 groups of rats, 72 h after acute KIR. (**H**–**M**) Representative IHC images (200×) for positively stained Wilm's tumor suppressor gene1 (WT-1) (gray color) in podocytes from 6 groups of rats, 72 h after acute KIR. (**N**) Quantification of total number of WT-1 positive cells in the kidney sections from 6 groups of rats, 72 h after acute KIR. *denotes statistical significance vs. other groups represented with different symbols (†, ‡, §, ¶), *p* < 0.0001. Scale bars in right lower corner represent 50 μm.

However, both FSP-1 and WT-1, which are predominantly expressed in kidney interstitials and podocytes, respectively showed opposite expression pattern to ZO-1 as analyzed by IHC (Figure 10).

### Status of dystroglycan and podocin expression on day 3 after acute KIR

**Figure 11:**
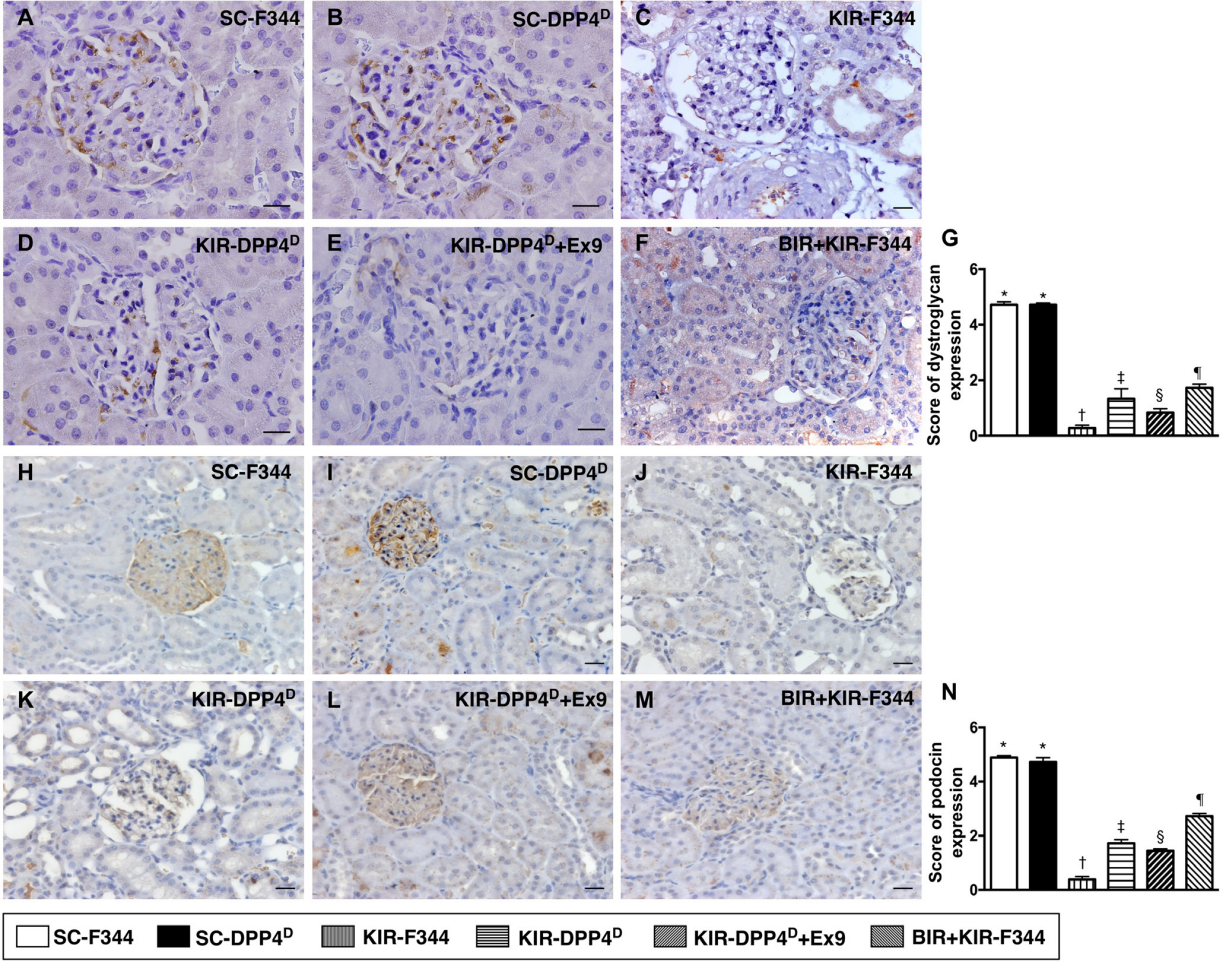
IHC analysis of dystroglycan and podocin on day 3 after acute KIR (**A**–**F**) Representative IHC images (400×) for positively stained dystroglycan (gray color) in podocyte foot process from 6 groups of rats, 72 h after acute KIR. (**G**) Quantification of total number of dystroglycan positive cells. (**H**–**M**) Representative IHC images (200×) for positively stained podocin (gray color) in podocytes from 6 groups of rats, 72 h after acute KIR. (**N**) Quantification of total number of podocin positive cells in kidney sections from 6 groups of rats, 72 h after acute KIR. *denotes statistical significance vs. other groups represented with different symbols (†, ‡, §, ¶), *p* < 0.0001. Scale bars in right lower corner represent 50 μm.

We demonstrated that dystroglycan, and podocin, two components of podocyte foot processes, were highly expressed in SC-F344 and SC-DPP4^D^ rats followed by progressively decreased levels in BIR-KIR-F344, KIR-DPP4^D^, DPP4^D^-KIR- exendin-9-39 rats and were lowest in KIR-F344 rats (Figure 11).

## DISCUSSION

In this study, we investigated the role of DPP4 in acute KIR injury by using DPP knockout rats. We observed that baseline circulating GLP-1 levels were higher in DPP4^D^ rats compared to F344 rats. After KIR, GLP-1 levels were higher in the KIR-F344 group compared to SC-F344, but lower compared to KIR-DPP4^D^ and BIR-KIR-F344 groups. This suggested that DPP4 deficiency and remote ischemic preconditioning protected kidneys against acute IR injury.

Previously, we showed that sitagliptin (DPP4 inhibitor) or exendin-4 (GLP-1 analogue) protected architectural integrity of the kidneys and renal function during acute KIR injury [14, 15]. However, the role of DPP4 during acute kidney IR injury was not directly investigated, especially in regard to GLP-1 [14, 15]. In this study, we showed that the circulating levels of creatinine and BUN and urine protein to creatinine ratio and anatomical/histopathological injury (H&E, IHC and IF showing kidney morphology and kidney injury score) were substantially higher in KIR-F344 animals than in SC-F344 animals, but reduced in KIR-DPP4^D^ animals. This corroborated our previous findings that DPP4 influenced the extent of kidney damage during IR injury [14, 15]. These findings highlight the therapeutic potential of the DPP4 inhibitors and GLP-1 analogues during AKI, especially in type 2 DM patients.

Remote ischemic preconditioning has been shown to protect other organs/tissues from IR injury [29–31]. Remote ischemic preconditioning involves complex signaling and interactions that activate ATP-dependent potassium channels, adenosine and PKC, release autacoids such as adenosine and bradykinin, generate neurohormones, nitric oxide and chemokines (such as hypoxia-inducible factor-1α), open mitochondrial K^+^-ATP-channels and elicit GSK3β and STAT-3 signaling that attenuate opening of the mitochondrial permeability transition pore [29–31]. In this study, we show that remote ischemic preconditioning by small BIR protects kidneys against acute KIR injury. The renal function and kidney architectural integrity was better preserved in BIR-KIR-F344 rats compared to KIR-DPP4^D^ rats.

The signal transduction pathways in ischemic pre-, post-, and remote conditioning that protect tissues/organs against acute IR are highly complex [30]. In the present study, circulating GLP-1 levels were higher in BIR-KIR-F344 animals at 3 to 6 hrs after acute KIR compared to the KIR-F344 group. Also, the circulating GLP-1 levels increased rapidly during the acute phase in BIR-KIR-F344 rats suggesting that the gastrointestinal-digestive tract was a major source of GLP-1 in response to ischemic stimulation as previously suggested [30].

Numerous studies have demonstrated that AKI or acute KIR injury generates oxidative stress and reactive oxygen species [13–15], elicits cellular apoptosis [11, 13–15], and activates complement and inflammation [10–14, 27]. These factors damage the kidney function and its anatomical structure [13–15, 35]. In our study, the acute KIR rats showed increased inflammation, oxidative stress, DNA damage and apoptosis consistent with previous studies [10–15]. The extent of functional and architectural damage to the kidneys in KIR-F344 rats was significantly higher compared to SC-F344, but lower in KIR-DPP4^D^ and BIR-KIR-F344 rats.

In a previous study, we demonstrated that acute KIR substantial damaged the kidney ultrastructure [15]. In this study, we confirmed that KIR substantially damaged the kidney ultrastructural integrity (components of podocyte foot process in glomeruli, renal tubule and extracellular matrix) in KIR-F344 rats compared to SC-F344 animals, but was better preserved in KIR-DPP4^D^ and BIR-KIR-F344 rats. This suggested that DPP4 deficiency and remote ischemic preconditioning preserved renal function during acute kidney injury.

We also demonstrated increased expression of antioxidant proteins like NQO 1, HO-1 and SOD-1 and GLP-1 receptor in the kidney tissues of KIR-DPP4^D^ and BIR-KIR-F344 rats compared to the sham controls and KIR-F344 rats. These findings suggested that DPP4 deficiency and remote ischemic preconditioning protected kidneys from the damage by ischemic stimulation and IR injury. These findings support and improve our understanding of mechanisms that enable protective ischemic conditioning against acute IR injury in tissues/organs [30].

**Figure 12:**
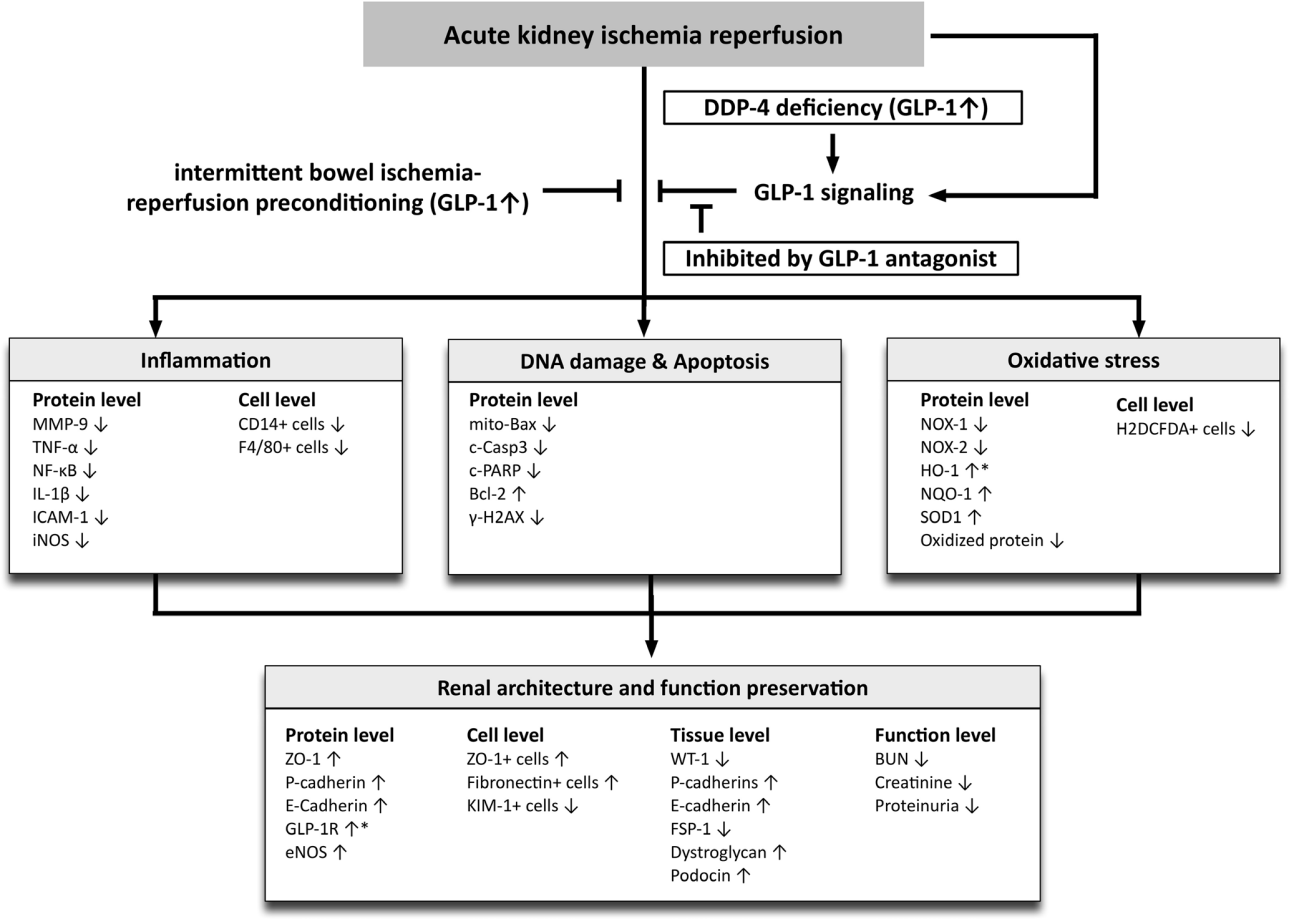
Proposed mechanisms underlying the positive therapeutic effects of DPP4 deficiency and small bowel ischemia-reperfusion (IR) preconditioning against acute kidney IR injury GLP-1 = glucagon like peptide 1; DDP4 = dipeptidyl peptidase 4 deficiency; MMP = matrix metalloproteinase; TNF = tumor necrosis factor; NF = nuclear factor; IL = interleukin; ICAM = intercellular adhesion molecule; iNOS = inducible nitric oxide synthase; mito = mitochondrial; c-Casp = cleaved caspase 3; c-PARP = cleaved protein expression of Poly (ADP-ribose) polymerase; HO = heme oxygenase; NQO 1 = NAD(P)H quinone dehydrogenase 1; eNOS = endothelial nitric oxide synthase; KIM = kidney injury molecule; WT-1 = Wilm's tumor suppressor gene 1; FSP-1 = fibroblast specific protein 1; BUN = blood urea nitrogen.

This study has limitations that need to be highlighted. First, the study period was only 72 h and therefore long term impact of DPP4 deficiency and remote ischemic preconditioning were not addressed. Second, although the small BIR procedure was used to elicit remote ischemic preconditioning in rats and shown to protect the kidneys from IR injury, such a method is not currently feasible for human patients. Third, although we identified changes in many signaling and functional entities in response to acute kidney injury as well as their status in DPP4 deficient or remote ischemic preconditioning conditions, the exact interplay of the various pathways and their relationship to renal oxidative stress, inflammation or cellular apoptosis are still not clear. Figure 12 summarizes probable protective mechanisms in the DPP4^D^ rat kidneys and remote ischemic preconditioning against acute KIR. Fourth, although BIR was comparable to DPP4 deficiency in protecting the kidney from acute IR injury, it wasn't clear if increased GLP-1 mediated the protective effects of BIR.

In conclusion, our study demonstrates that DPP4 deficiency and remote ischemic preconditioning protect kidneys from acute IR injury by elevating circulating GLP-1 levels and represent potential therapeutic targets for acute kidney injury, especially in type 2 diabetic patients.

## MATERIALS AND METHODS

### Experimental animals

The animal experiments were conducted according to approved protocols by the Animal Care and Use Committee at Kaohsiung Chang Gung Memorial Hospital (Affidavit of Approval of Animal Use Protocol No. 2015032402) and executed according to the Guide for the Care and Use of Laboratory Animals, 8th edition (NRC 2011). Pathogen-free, adult male (*n* = 32) and dipeptidyl peptidase 4 (DPP4) mutant (*n* = 16) Fischer 344 rats weighing 300–320 g were obtained from Charles River Technology (BioLASCO Taiwan Co. Ltd., Taiwan). The animals were housed in an AAALAC-approved animal facility in our hospital with controlled temperature and light cycles (24°C and 12/12 h light/dark cycles).

### Acute kidney ischemia-reperfusion (IR) protocol and animal grouping

The acute kidney IR was performed according to previously published protocol [15, 35]. Briefly, animals were anesthetized with 2% isoflurane and placed supine on a warming pad at 37°C for midline laparotomies. The sham control (SC) animals underwent laparotomy only. Acute IR injury of both kidneys was induced by clamping the renal pedicles of the rats with non-traumatic vascular clips for 60 minutes followed by reperfusion for 72 h. The animals were then euthanized and the kidneys were harvested for further experiments. Left kidney was used for H&E staining, immunofluorescence (IF) and immunohistochemistry (IHC) and the right kidney was used for western blotting.

The rats were categorized into six groups (*n* = 8 group): (1) sham control F344 (SC-F344) with laparotomy without kidney IR (KIR); (2) sham control DPP4 mutant rats (SC-DPP4^D^); (3) kidney IR in F344 rats (KIR-F344); (4) kidney IR in DPP4 mutant rats (KIR-DPP4^D^); (5) KIR-DPP4^D^ with intraperitoneal injection of 10 mg/kg exendin-9-39 at 30 minutes and days 1–3 after KIR; and (6) small bowel IR in F344 rats with KIR (BIR-KIR-F344). Exendin-9-39 is an antagonist of exendin 4, a hypoglycemic agent and a GLP-1 analog used for type II diabetes mellitus. The dosage of exendin-9-39 was based on our previous study [14].

### Remote ischemic preconditioning by intermittent small bowel IR

The small bowel IR injury was performed according to previously published protocol [36]. Briefly, the small BIR injury in group 6 rats involved clamping the superior mesenteric artery for 5 mins followed by reperfusion for 5 mins and repeating the procedure 6 times. The acute kidney IR procedure was performed immediately after finishing the small BIR procedure.

### Estimating kidney injury scores after acute KIR

Histopathology scoring was performed on kidney samples isolated on day 3 after KIR and were assessed blind as previously described [13–15]. Briefly, the kidney specimens were fixed in 10% buffered formalin and embedded in paraffin. Then, 5 μm sections were cut and stained with hematoxylin and eosin (H&E) and observed by light microscopy. The scoring system involved grading the extent of tubular necrosis, loss of brush border, cast formation and tubular dilation in 10 randomly chosen non-overlapping fields (200×) for each rat kidney sample [32]. The scores were 0 (0%), 1 (≤ 10%), 2 (11–25%), 3 (26–45%), 4 (46–75%), and 5 (≥ 76%).

### Estimation of blood urea nitrogen and creatinine and urine protein to creatinine ratio after acute KIR

Blood samples were collected from the rats in all 6 groups before and 72 h after IR to measure changes in serum creatinine and urea nitrogen.

To determine the ratio of urine protein to creatinine, each animal was placed in a metabolic cage (DXL-D, 190 mm × 290 mm × 550 mm, Suzhou Fengshi Laboratory Animal Equipment Co. Ltd., Mainland China) for 24 h with free access to food and water. Urine was collected for 24 h before and 72 h after IR.

### IHC and IF staining

The IHC and IF staining was performed according to previously published protocols [13–15]. For IHC and IF staining, rehydrated paraffin sections were treated with 3% H_2_O_2_ for 30 minutes and then incubated with Immuno-Block reagent (BioSB, Santa Barbara, CA, USA) for 30 minutes at room temperature. Then, the sections were incubated with the following primary antibodies: zonula occludens-1 (ZO-1; 1:300, Abcam, Cambridge, MA, USA), Wilm's tumor suppressor gene 1 (WT-1; 1:1000, Abcam, Cambridge, MA, USA), kidney injury molecule (KIM-1;1:200, R&D systems, Minneapolis, MN, USA), fibroblast specific protein (FSP-1; 1:200, Abcam, Cambridge, MA, USA), P-cadherin (1:100, Novus, Littleton, CO, USA), podocin (1:100,Sigma, St. Louis, Mo, USA), dystrophin (1:100, Abcam, Cambridge, MA, USA), fibronectin (1:200, Abcam, Cambridge, MA, USA), dystroglycan (1:50, Abcam, Cambridge, MA, USA), synaptopodin (1:100, Santa Cruz, CA, USA), CD14 (1:50, Santa Cruz, CA, USA), CD68 (1:100, Abcam, Cambridge, MA, USA); sections incubated with irrelevant antibodies served as controls. Three kidney sections were analyzed from each rat. For quantification, three randomly selected high power fields (HPFs; 200× for IHC; 400× for IF) were analyzed in each section. The mean number of positively-stained cells per HPF for each animal was determined by summation of all numbers divided by 9.

### Western blot analysis

Western blot analysis was performed according to previous published protocols [13–15]. Briefly, 50 μg of total kidney protein extract from different groups of rats were separated by SDS-PAGE using acrylamide gradients. Then, the separated proteins were transferred electrophoretically to a polyvinylidene difluoride (PVDF) membrane (Amersham Biosciences, Amersham, UK). Non-specific binding was blocked by incubating the membrane in 5% nonfat dry milk in T-TBS (TBS containing 0.05% Tween 20) overnight. Then, the membranes were incubated for 1h at room temperature with primary antibodies against matrix metalloproteinase (MMP-9; 1:3000, Abcam, Cambridge, MA, USA), tumor necrosis factor (TNF-α; 1:1000, Cell Signaling, Danvers, MA, USA), nuclear factor (NF-κB; 1:600, Abcam, Cambridge, MA, USA), interleukin (IL-1β; 1:1000, Cell Signaling, Danvers, MA, USA), intercellular adhesion molecule (ICAM-1; 1:1000, Abcam, Cambridge, MA, USA), inducible nitric oxide synthase (iNOS; 1:200, Abcam, Cambridge, MA, USA), cleaved caspase 3 (1:1000, Cell Signaling, Danvers, MA, USA), cleaved PolyADP-ribosepolymerase (c-PARP; 1:1000, Cell Signaling, Danvers, MA, USA), mitochondrial Bax (1:1000, Abcam, Cambridge, MA, USA), γ-H2AX (1:1000, Cell Signaling, Danvers, MA, USA), endothelial nitric oxide synthase (eNOS; 1:1000, Abcam, Cambridge, MA, USA), heme oxygenase-1 (HO-1; 1:500, Cell Signaling, Danvers, MA, USA), glucagon like peptide 1 receptor (GLP-1R; 1:1000, Abcam, Cambridge, MA, USA), superoxide dismutase (SOD; 1:2000, Abcam, Cambridge, MA, USA), NAD(P)H dehydrogenasequinone 1 (NQO 1; 1:1000, Abcam, Cambridge, MA, USA), NOX-1 (1:1500, Sigma, St. Louis, Mo, USA), NOX-2 (1:750, Sigma, St. Louis, Mo, USA), and actin (1: 10000, Chemicon, Billerica, MA, USA). Horseradish peroxidase-conjugated anti-rabbit immunoglobulin IgG (1:2000, Cell Signaling, Danvers, MA, USA) was used as a secondary antibody for 1h at room temperature. Then, after washing, the blots were developed and visualized by enhanced chemiluminescence (ECL; Amersham Biosciences, Amersham, UK) and exposed to Biomax L film (Kodak, Rochester, NY, USA). ECL signals were quantitated using Labwork software (UVP, Waltham, MA, USA).

### Oxyblot oxidized protein detection

Oxidative stress was assessed according to previously published protocols [13–15] using the Oxyblot Oxidized Protein Detection Kit (S7150, Chemicon, Billerica, MA). First, we DNPH derivatized 6 μg total rat kidney protein for 15 minutes according to the manufacturer's instructions. Then, the DNPH derivatized proteins were electrophoresed on a 12% SDS-PAGE followed by electrophoretic transfer of proteins to nitrocellulose membranes. After blocking, the membranes were incubated with primary anti-DNP1 antibody (1:150) for 2 h followed by incubation with secondary antibody (1:300) for 1 h at room temperature. After washing repeatedly, the blots were developed and visualized by enhanced chemiluminescence (ECL; Amersham Biosciences, Amersham, UK) and exposed to Biomax L film (Kodak, Rochester, NY, USA). ECL signals were quantified using Labwork software (UVP, Waltham, MA, USA). A standard control was loaded on each gel as reference for oxyblot protein analysis.

### Assessment of ROS by H2DCFDA

We assessed reactive oxygen species (ROS) according to previously published protocols [34]. Thirty minutes prior to the end of the study, the rats were anesthetized with 2% isoflurane and intravenously administered 150 μg of CM-H2DCFDA (Life Technologies Molecular Probes; 100 μg of H2DCFDA was dissolved in 200 μl PBS). After 30 minutes, the kidneys were harvested and immediately frozen in liquid nitrogen. Then, 5 μm thick cryostat sections were cut at −20°C and the serial frozen sections were fixed with 4% paraformaldehyde in phosphate buffered saline at 4°C for 5 minutes. After washing the sections with PBS, they were co-stained with DAPI and analyzed by fluorescence microscopy.

All sections were examined under a fluorescent microscope (200×). The fluorescence and gray photos were assessed by DP controller 2.1.1.183 (Olympus). The gray photos were used to measure fluorescence intensity with Image J 1.37v (National Institutes of Health, USA). Three gray photos from three sections were randomly analyzed for each animal. The baseline fluorescence intensity (BFI) [arbitrary unit/200 × high-power field (HPF)] was determined by comparing the area of increased fluorescence intensity (IFI) with the area in the kidney without H_2_DCFDA. Six BFI areas were measured from each gray photo. The mean IFI and BFI were calculated from three randomly chosen BFI areas. The relative fluorescence intensity was determined from the ratio of IFI to BFI.

### Statistical analysis

Quantitative data was expressed as mean ± SD. Statistical analysis was performed by ANOVA followed by Bonferroni multiple-comparison post hoc test with the SAS statistical software for Windows version 8.2 (SAS institute, Cary, NC). A *P* < 0.05 was considered statistically significant.
